# State of the art of cervical cancer treatment in rare histologies

**DOI:** 10.3389/fonc.2024.1386294

**Published:** 2024-06-28

**Authors:** Eder Alexandro Arango-Bravo, Tatiana Galicia-Carmona, Lucely Cetina-Pérez, Celia Beatriz Flores-de la Torre, María Isabel Enríquez-Aceves, José Antonio García-Pacheco, Eva María Gómez-García

**Affiliations:** ^1^ Medical Oncology Department, National Institute of Cancerology (INCan), Mexico City, Mexico; ^2^ Clinical Investigation Department, National Institute of Cancerology (INCan), Mexico City, Mexico; ^3^ Oncology Department, Campeche State Oncology Center, Campeche, Mexico; ^4^ Oncology Department, Regional Instituto de Seguridad y Servicios Sociales de los Trabajadores del Estado (ISSSTE) Hospital León, León de los Aldama, Guanajuato, Mexico; ^5^ Sistema Nacional de Investigadores (SNI), National Council of Science and Technology (CONACYT), Mexico City, Mexico; ^6^ Oncology Department, Metepec Cancer Treatment Center, Metepec, Estado de México, Mexico

**Keywords:** cervical cancer, neuroendocrine carcinoma of the cervix, gastric type adenocarcinoma, adenosquamous adenocarcinoma, glassy cell adenocarcinoma, rare histologies of cervical cancer

## Abstract

The objective of this review is to summarize the current scientific evidence to formulate clinical recommendations regarding the classification, diagnostic approach, and treatment of rare histological subtypes of cervical cancer; neuroendocrine carcinoma, gastric-type mucinous adenocarcinoma, and glassy cell adenocarcinoma. These histological subtypes are generally characterized by their low frequency, aggressive biological behavior, certain chemoradioresistance, and consequently, high recurrence rates with a deleterious impact on survival. Molecular studies have identified several associated mutations in neuroendocrine carcinoma (PIK3CA, MYC, TP53, PTEN, ARID1A, KRAS, BRCA2) and gastric-type adenocarcinoma (KRAS, ARID1A, PTEN) that may serve as molecular targets. While adenocarcinomas are typically treated and classified based on squamous histology across early, locally advanced, and advanced stages, the treatment strategies for neuroendocrine carcinomas in early stages or locally advanced cases differ, particularly in the sequencing of administering chemotherapy, chemoradiotherapy, or surgery. The chemotherapy regimen is based on etoposide plus cisplatin (EP). Unlike squamous cell carcinomas, immune checkpoint inhibitors are yet to establish a standard role in the treatment of recurrent neuroendocrine carcinomas due to the absence of clinical trials. Regarding glassy cell adenocarcinomas and gastric-type adenocarcinoma, the potential use of immunotherapy in advanced stages/disease requires further evaluation through international collaborations, given the limited number of cases.

## Introduction

Cervical cancer (CC) is the fourth among global women’s malignancies. Despite successful screening and vaccination in high-income countries, its incidence and mortality surge in middle- and low-income nations. In Mexico, it stands as the second leading cause of cancer-related morbimortality in women (1, 2). The main histology is squamous, followed by endocervical adenocarcinoma. Currently, carcinomas and their precursor lesions are classified by their association with Human Papillomavirus (HPV) for prognostic and predictive purposes (3, 4). This review focuses on rare and aggressive adenocarcinoma subtypes: gastric-type, adenosquamous, and neuroendocrine carcinoma. Treatment recommendations rely on limited evidence from retrospective studies, case series, reports, or expert opinions.

## Methods

To obtain the information, the keywords “intervention”, “control”, and “results” were used to find articles that included rare subtypes of cervical cancer, endocervical adenocarcinoma, gastric-type adenocarcinoma, clear cell carcinoma of the cervix, glassy cell carcinoma, neuroendocrine cervical carcinoma. The search was conducted using the database browsers MEDLINE, PubMed, EMBASE, and the Medscape database, combining the following terms: “systemic treatment of neuroendocrine carcinomas of the cervix,” “treatment of adenocarcinoma of the cervix”, “clear cell variety”, “gastric type”, “adenosquamous (glassy cells).” Only articles published in the last 15 years and in English were included to ensure the most up-to-date collection of scientific evidence.

All authors participated in the review and data extraction of the original manuscripts. The first author was contacted to obtain articles for which the final version was not available online, ensuring that no information was lost. Finally, the articles underwent a thorough review, and the information was processed according to the GRADE system. The evidence was classified, and recommendations were made based on the grade of the evidence.

## Histopathological classification of cervical cancer

Squamous cell carcinoma (SCC), or epidermoid carcinoma, constitutes 75-80% of all CC cases, primarily linked to HPV (90%). Endocervical adenocarcinoma (EAC) constitutes 20-25%, with recent findings indicating 10-15% are not associated with high-risk HPV types. Rare histologies include neuroendocrine carcinoma (0.9%-1.1%), glassy cell, and gastric-type adenocarcinomas (3%), all associated with poor prognosis, and are independent to HPV (5).

In 2020, the World Health Organization (WHO) updated the genital tumor classification, emphasizing the HPV association (5, 6).

## Rare histologies of cervical cancer

### Endocervical adenocarcinomas

According to the 2020 WHO classification, this histological subgroup is divided into two subtypes: those associated with Human Papillomavirus (HPV-A) and those independent (HPV-I). HPV-A adenocarcinomas include three categories: the usual type (representing 80%, with villoglandular and micropapillary, variants); the mucinous type (intestinal, signet ring cells, and stratified invasive mucinous carcinoma); and the not otherwise specified (NOS) adenocarcinoma (7–9). Distinguishing usual from endometrioid adenocarcinoma involves assessing the expression of estrogen and progesterone receptors, p16, and p53 status (7).

On the other hand, HPV-I adenocarcinomas comprise gastric-type, clear cell, endometrioid, mesonephric, and NOS types. Their etiology is still unknown, but several associated molecular alterations have recently been demonstrated, including mutations in the *PIK3CA*, *KRAS*, and *PTEN* genes, as well as in members of the PI3K/Akt/mTOR signaling cascade. Some of these mutations have a predictive and prognostic value, considering them high-grade histological groups (10–12).

It is worth mentioning that evidence indicates that clear cell carcinoma has worse disease-free survival and overall survival compared to HPV-A adenocarcinomas, with survival rates similar to gastric-type adenocarcinomas (10–12).

### Treatment of rare variants of cervical adenocarcinoma

#### Gastric-type mucinous adenocarcinoma

Cervical adenocarcinoma typically follows squamous histology guidelines in staging and treatment, as clinical trials encompass both histological populations. However, gastric-type mucinous adenocarcinoma, constituting 10% globally (20-25% in Japanese population), is a distinct variant within this classification (12, 13).

Yevgeniy S. Karamurzin et al.’s retrospective study, based on data from three institutions, compared gastric-type with usual HPV-A adenocarcinoma. Gastric-type cases often presented at later stages; only 41% were early-stage (FIGO [International Federation of Gynecology and Obstetrics] IA1 - IB2), while 89% of usual adenocarcinoma cases were. For stages II-IV, 59% of gastric-type cases were diagnosed at advanced stages, in contrast to 11% in the usual group. Gastric-type’s metastatic pattern involved lymph nodes, annexes, omentum, intestines, peritoneum, diaphragm, abdominal wall, bladder, vagina, appendix, and central nervous system, resulting in a 42% 5-year overall survival rate, significantly lower than the 91% rate for usual adenocarcinoma. This emphasizes the aggressiveness of gastric-type adenocarcinoma, characterized by advanced stage presentation, increased metastasis, and a distinct pattern of spread, leading to a poorer prognosis compared to the usual type (14).

##### Is differential treatment needed for gastric-type endocervical adenocarcinoma compared to usual-type endocervical adenocarcinoma?

Currently, no scientific evidence supports distinct treatment for mucinous gastric-type endocervical adenocarcinoma compared to usual adenocarcinoma. Standard approaches include surgery with adjuvant therapy for early stages and chemoradiotherapy for locally advanced cases. Platinum-based chemotherapy plus bevacizumab is recommended for advanced disease. Altered genetic pathways identified through sequencing and immunohistochemistry may guide future personalized treatment (14). The accumulating evidence may or may not designate GAS as a high-risk factor for recurrence, potentially justifying more aggressive early-stage treatment.

##### Recommendations

• There is still no high-quality scientific evidence specific for this histological subtype, so recommendations are indirect based on the management of UEA.
• No differential treatment between UEA and GAS; standard early-stage treatment involves surgery with adjuvant therapy for intermediate/high-risk factors (low).
• Locally advanced stages (IB to IIIC2) are treated with concurrent chemoradiotherapy using weekly cisplatin (low).
• For advanced or recurrent cases without local treatment options, preferred therapy includes chemotherapy (cisplatin/paclitaxel) plus bevacizumab ± pembrolizumab (for PD-L1 positive tumors) (low).
• Recurrent disease lacks a standard treatment; single-agent chemotherapy (topotecan, vinorelbine, gemcitabine, pemetrexed, docetaxel) or cemiplimab monotherapy for immunotherapy-naive patients are options (moderate).

#### Clear cell adenocarcinoma

Comprising 3-4% of cervical adenocarcinomas, the etiology of clear cell adenocarcinoma remains unclear. It is considered highly invasive, unrelated to HPV infection, with potential links to diethylstilbestrol exposure (15–17), cervical endometriosis, oral contraceptive use, and HIV infection. Three main morphological patterns exist: tubulocystic (most common), papillary (least common), and solid. Immunohistochemical staining aids in differentiation, with positive Napsin A and HNF1-beta, negative progesterone and estrogen receptors (PR, ER), negative p16, and normal p53 in most cases, distinguishing it from the endometrial clear cell variety (18, 19).

##### Treatment for clear cell carcinoma of the cervix

Clear cell carcinoma is treated similarly to adenocarcinoma and squamous cell carcinoma. Standard surgical treatment for early FIGO stages IB or IIA involves radical hysterectomy and pelvic lymphadenectomy. Stages IIB to IIIB are managed with external radiotherapy and concurrent platinum-based chemotherapy. A recent study comparing radical trachelectomy to radical hysterectomy for stage IB1 cervical carcinoma in young women showed comparable survival outcomes, supporting fertility-preserving surgery for early-stage cancer (20). However, caution is advised due to limited specific information on this subtype.

While ovarian clear cell carcinomas often have an unfavorable prognosis, clear cell carcinoma of the cervix does not consistently show a worse prognosis than squamous cell carcinoma. Prognostic factors include FIGO stage, parametrial involvement, cervical stromal extent, positive surgical margins, tumor diameter, and lymphatic vascular space involvement. Recurrence tends to occur in the pelvis, para-aortic lymph nodes, and distant sites, with a median time to recurrence of 8 months for stages I and II (21). Common sites of recurrence include the pelvis, para-aortic lymph nodes, and distant sites. Clear cell carcinomas of the lower genital tract have a greater tendency to recur late and develop metastases in distant sites more frequently than squamous cell carcinomas (22). Extrapelvic recurrence sites may include the lungs, liver, and bone (23).

#### Glassy cell carcinoma

Glassy cell carcinoma, previously considered a distinct histological variant, now falls under adenosquamous carcinoma, constituting around 2% of cervical carcinomas (24). Typically diagnosed around the age of 46.9 years, it often occurs in young patients and may be associated with pregnancy. Despite its previous classification, the 2020 WHO categorizes it as a variety of adenosquamous carcinoma. Glassy cell carcinoma carries a poor prognosis marked by rapid proliferation, high recurrence risk, and metastasis. There is a potential association with high-risk HPV (16, 18, and 32) (25). Histologically, it features large undifferentiated non-keratinizing cells with prominent membranes, eosinophilic cytoplasm, large nuclei, and nucleoli, showing immunoreactivity to p16 (26).

##### Glassy cell carcinoma treatment

Due to its rarity and limited studies, the treatment of glassy cell carcinoma has predominantly followed guidelines for the squamous variety. Case series consistently report poor responses to standard treatments such as surgery, radiotherapy, and chemotherapy compared to other carcinoma types.

A meta-analysis of 292 patients indicated a 5-year survival of 54.8% and an average overall survival of 25 months, noting that patients in stage I treated with surgery alone had a recurrence rate of 32% compared to those who received surgery plus radiotherapy, which was 21% (27). However, these results, where a low percentage of survival is observed, may be influenced by sub-staging. Recent observations suggest that early-stage treatment should be multimodal due to the aggressive nature of glassy cell carcinoma.

In a study of 20 cases with a mean 28-month follow-up, stage I disease-free survival was 93%. Of these, 14 stage IB patients underwent total hysterectomy with pelvic lymphadenectomy, of which 10 received adjuvant therapy using weekly paclitaxel/carboplatin for 4 cycles, showing improved recurrence-free survival. However, this study has a small sample size (28).

Early-stage treatment follows international standards for squamous varieties. For tumors <2 cm, radical hysterectomy with pelvic lymphadenectomy plus resection of common, internal, and external iliac nodes, as well as the obturator nerve is recommended (29).

Young, nulliparous patients may consider radical vaginal trachelectomy with pelvic lymphadenectomy, but candidacy depends on tumor size (≤2 cm, due to high risk of ectopic involvement and recurrence with tumors larger than 2 cm), determined by magnetic resonance imaging (30).

Adjuvant therapy is based on risk groups. For stages IIB-III, concurrent radiotherapy with cisplatin-based chemotherapy aligns with international guidelines, showing a 67% recurrence-free survival benefit at 28 months. In clinical stage IVB, standard treatment is platinum-based chemotherapy plus paclitaxel ± bevacizumab + pembrolizumab (31).

##### Recommendations (clear cells and glassy cells carcinomas)

###### Early disease

• Abdominal radical hysterectomy plus bilateral pelvic lymphadenectomy followed by adjuvant therapy is the standard treatment. Administer concurrent radiotherapy with cisplatin-based chemotherapy for high-risk patients and radiotherapy alone for the intermediate group (high).
• Expert recommendations suggest administering the cisplatin/paclitaxel doublet concurrently with radiotherapy for 4 cycles in the glassy cell variety (very low).

###### Locally advanced disease

• Offer concurrent chemoradiotherapy with weekly cisplatin (high).
• No recommendation for neoadjuvant or adjuvant treatment outside clinical trials due to the lack of prospective studies.

###### Advanced disease

• For stage IVB or recurrent disease without local treatment options, prefer platinum-based chemotherapy plus paclitaxel ± bevacizumab + pembrolizumab (for PD-L1+ tumors) (low).
• In the context of recurrent disease, consider chemotherapy options like monotherapy with topotecan, vinorelbine, gemcitabine, pemetrexed, and docetaxel (moderate).

### Neuroendocrine carcinomas of the cervix

Neuroendocrine small cell carcinoma (NEC) of the cervix is a rare disease, representing less than 2% of all invasive cervical cancers (32). In a database series from 1997 to 2003 by the Surveillance, Epidemiology, and End Results (SEER) in the United States, the average annual incidence was 0.06 per 100,000 women, compared to 6.6 and 1.2/100,000 women for squamous cell carcinoma and adenocarcinoma, respectively. The average age at diagnosis is 47 years (4, 33). The established causal factor for the disease is high-risk HPV infection, supported by a meta-analysis of over 143 studies, demonstrating an association of up to 85% between small cell cancers and HPV infection. Of these, 78% were HPV 16 and/or 18(+), 51% were solely HPV 18(+), and 10% were solely HPV 16(+) (34). Unlike small cell lung carcinoma, smoking has not been established as a risk factor for this subtype; however, it has been linked to a worse prognosis (35). According to the WHO, there is an updated 5^th^ classification system for gynecological neuroendocrine neoplasms, summarized in **Table 1**. It is worth mentioning that the neuroendocrine carcinomas addressed in this review are considered, by definition, high-grade neoplasms (36). Thus, the most common subtype of NECs is the small cell carcinoma, representing 80%, followed by large cell carcinoma (12-15%), with the remaining less than 8% (37). The histopathology diagnosis requires both morphological characteristics and the identification of neuroendocrine markers by immunohistochemistry (IHC), specifically using markers such as chromogranin A, synaptophysin, neuron-specific enolase (NSE), CD56 (neural cell adhesion molecule), and Ki-67 for histological grading, and allowing differentiation from neuroendocrine tumors (NETs) (38).

**Table 1:** General characteristics, prognosis, and treatment of rare histologies of cervical cancer.

WHO 2020 Classification	Histologic grade	Age	IHC stains	Prognostic information	Treatment
**Squamous Cell Carcinoma** *HPV-associated SCC *SCC, not associated with HPV *SCC, NOS (Not Otherwise Specified)	Grade 1-3	50-60 years	P16 (+) or (-), Cytokeratin 7, p63, and p40 (+)	Dependent on clinical stage Better prognosis than rare histologies.	- Surgery (early stages) and consider adjuvant therapy based on clinical-pathological factors. - Chemoradiotherapy concurrent with cisplatin (LAD) - Palliative chemotherapy (advanced disease) ± immunotherapy
**Endocervical adenocarcinoma** HPV-associated (HPV-A) ECA *Usual adenocarcinoma *Mucinous adenocarcinoma (NOS, intestinal, signet ring cell, ISMC) *Adenosquamous *Adenoid basal. *Mucoepidermoid *Adenocarcinoma NOS	Grade 2-3	40-60 years	P16 (+), ER and PR (-), p53 (wild-type or aberrant)	Better prognosis when associated with HPV	- Surgery (early stages) and consider adjuvant therapy based on clinical-pathological factors. - Chemoradiotherapy concurrent with cisplatin (LAD) - Palliative chemotherapy (advanced disease) ± immunotherapy
**Endocervical adenocarcinoma** HPV-independent (HPV-I) ECA *Gastric adenocarcinoma (40-50^a^) *Clear cell adenocarcinoma *Mesonephric adenocarcinoma *Endometrioid adenocarcinoma *Adenocarcinoma NOS	Grade 2-3	50-70 years	P16 (-), Napsin A (+), HNF1 (+), ER and PR (-)	Worse prognosis than squamous and HPV-associated adenocarcinomas.	-Surgery (early stages) -Chemoradiotherapy with concurrent cisplatin (LAD) -Palliative chemotherapy (advanced disease) consider (CapeOx) scheme, review recommendations.
*Neuroendocrine Carcinoma (NEC) *Small Cells *Large Cells *Combined NEC with Small Cells *Combined NEC with Large Cells	3 (high grade) 3 (high grade) 3 (high grade) 3 (high grade) 3 (high grade)	30-60 years	-Synaptophysin (+) -Neuronal-specific enolase (+) -Chromogranin (+) -CD 56 (+) -Ki-67	Poor prognosis, aggressive histologies. Good sensitivity to cisplatin	-Surgery plus adjuvant therapy with EP regimen (early disease) -Chemoradiotherapy with EP followed by adjuvant therapy (LAD) -Palliative chemotherapy in advanced disease.

SCC, Squamous Cell Carcinoma or Epidermoid Carcinoma; ECA, Endocervical adenocarcinoma; NOS, Not Otherwise Specified; HPV, Human Papillomavirus; ISMC, Invasive Stratified Mucinous Carcinoma; LAD, locally advanced disease; IHC, immunohistochemistry; ER, estrogenic receptors; PR, progesterone receptors; NEC, neuroendocrine carcinoma; EP, etoposide/cisplatin.

#### What would be the ideal treatment for neuroendocrine cervical carcinoma by clinical stages?

##### Treatment in early stages (stage IA1 to IIA FIGO)

-*Surgery vs. radiotherapy in early disease:* There are no prospective data comparing surgery followed by chemotherapy versus definitive chemoradiation in patients with resectable early-stage NEC. However, several retrospective analyses have evaluated the utility of primary radical surgery followed by adjuvant treatment. The study by JM Lee et al., evaluating 68 patients with small cell NEC in stage IB-IIA who underwent radical surgery, reported a 2-year overall survival median of 64.6% and a 5-year median of 46.6%. Univariate and multivariate analyses suggested that adjuvant chemotherapy tended to improve overall survival (35). Another study by PJ Hoskins et al., where 3-year recurrence-free survival rates were 80% in patients with early-stage small cell NEC (stage I-II) who received primary radiotherapy followed by platinum-based chemotherapy (39). A retrospective study by Chen TC et al., from 11 hospitals in Taiwan, reported a 5-year overall survival rate of 78% for primary radiotherapy with at least five cycles of platinum-based chemotherapy, compared to 46% for primary surgery alone in patients with early-stage NEC (40). Conversely, Cohen et al., identified that 5-year overall survival for stages I-IIA improved (38% vs. 24%) in patients treated with radical hysterectomy plus adjuvant chemotherapy and/or chemoradiotherapy compared to those who did not receive these treatments (41). Based on the conflicting results of multiple retrospective studies, the role of surgery for early-stage neuroendocrine tumors appears uncertain; however, surgery alone without adjuvant chemotherapy or radiotherapy is not recommended due to the high-grade histology and poor prognosis (35). Therefore, adjuvant chemotherapy is needed even in patients with early-stage disease.
-Adjuvant chemotherapy: Adjuvant chemotherapy is commonly used in patients with small cell NEC. Treatment approaches have been extrapolated from small cell lung cancer therapy. The combination of etoposide/cisplatin is the most frequently used adjuvant chemotherapy regimen in small cell NEC patients. A retrospective analysis by Zivanovic O et al., including patients with stages IA1-IB2 according to FIGO, showed that adjuvant chemotherapy in initial treatment significantly improved 3-year distant recurrence-free survival (83% vs. 0%, *p* = 0.03) and 3-year overall survival (83% vs. 20%, p = 0.36) (42). Adjuvant chemotherapy after surgery improved disease-free survival and reduced extrapelvic recurrences with an odds ratio of 0.37 (*p* = 0.047) (43). Regarding the number of cycles of adjuvant chemotherapy, receiving at least five cycles was associated with better 5-year recurrence-free survival compared to other regimens (68% vs. 21%, *p* < 0.001) (44).
-Adjuvant Radiotherapy: A retrospective analysis by Shane R. Stecklein et al., documented disease-free survival in patients who received adjuvant radiotherapy compared to those treated with surgery alone (9.0 months vs. 18.0 months, *p* = 0.49). Similarly, overall survival was 23.0 months for women who received adjuvant radiotherapy vs. 38.0 months for those treated with surgery alone (*p* = 0.38). There were no differences in pelvic recurrence rates between women treated with surgery vs. adjuvant radiotherapy (44% vs. 57%, *p* = 0.61) (45). On the other hand, Wharton D. et al., in a retrospective analysis, found that early-stage small cell NEC patients who received platinum-based adjuvant chemotherapy likely had better survival than patients who received concomitant adjuvant chemoradiotherapy (5-year overall survival, 52.5% vs. 45.5% (46). However, in a multicenter Japanese study by Frumovitz et al., the administration of adjuvant radiotherapy was linked to a reduced risk of postoperative pelvic recurrence compared to patients who did not receive it (16% vs. 25%). While the difference did not reach statistical significance, there appears to be at least a trend towards improving local control of the disease (47). Although there seems to be a trend indicating that adjuvant chemotherapy plays a crucial role in the treatment of women with early-stage disease, there is conflicting data on the role of surgery followed by concurrent chemotherapy.

###### Recommendations

• Treatment for early-stage disease involves radical hysterectomy and lymphadenectomy followed by adjuvant chemotherapy with the EP scheme for 5 cycles (moderate).
• Alternative option considered is radical hysterectomy and lymphadenectomy followed by concurrent chemoradiotherapy with 2 cycles of EP followed by 4 cycles of adjuvant chemotherapy (low).

##### Treatment in locally advanced disease (IB3, IIA2, IIB, IIIA, IIIB, IIIC1, IIIC2, and IVA)

As known, concurrent chemoradiotherapy with cisplatin is the standard treatment for locally advanced disease in squamous and adenocarcinoma histologies. However, in NEC, the use of cisplatin monotherapy seems insufficient for disease control. Hence, the combination with cisplatin/etoposide or carboplatin/etoposide given concurrently with radiotherapy is the best treatment option with an adequate toxicity profile and manageable side effects (44). In a subanalysis by Pei Xuan et al., in stages IIB-IVB, concurrent chemoradiotherapy with the etoposide/cisplatin (E/P) scheme followed by chemotherapy with the E/P scheme for 5 cycles improved 5-year disease-free survival (63% vs. 13%, *p =* 0.025) and overall survival (75% vs. 17%, *p* = 0.016) compared to other treatments (48).

Another retrospective analysis by Tyler P. Robin et al., in patients with NEC who received cisplatin/etoposide, showed improved overall survival (59% vs. 44%) and lower recurrence rates (65% vs. 74%) compared to those who received cisplatin alone (49). The addition of brachytherapy, compared to external beam radiotherapy (EBRT) alone, improved median survival to 49 months vs. 22 months; however, there were no differences in overall survival between patients treated with neoadjuvant chemotherapy and those treated with chemotherapy and radiotherapy (HR=0.85, 95% CI=0.48-1.50) (49).

On the other hand, neoadjuvant chemotherapy as a treatment option in the context of locally advanced disease may be another alternative. A retrospective analysis by Giuseppe Caruso et al., evaluated the usefulness of radical surgery after neoadjuvant chemotherapy with the EP scheme, with response rates ranging from 50% to 80%. Progression-free survival and overall survival were 15.0 ± 30.6 months and 26.3 ± 36.4 months, respectively, comparable to results obtained with concurrent chemoradiotherapy (50).

###### Recommendations

The approach to the treatment of locally advanced disease must be multidisciplinary; hence, one of the following strategies could be followed.

• In locally advanced disease, concurrent chemoradiotherapy with the EP scheme (etoposide/cisplatin) for 2 cycles followed by 4 cycles of adjuvant EP is the best treatment alternative (moderate).
• According to expert recommendations, in cases of good tumor response, assessed by a multidisciplinary evaluation team using PET-CT after concurrent chemoradiotherapy with the EP scheme for 2 cycles, it is suggested to consider surgery followed by 2-4 cycles of adjuvant EP (low).
• Neoadjuvant chemotherapy with 2-3 cycles of EP, followed by radical surgery (after imaging response evaluation), as opposed to concurrent chemoradiotherapy with 2 cycles, could be a treatment option in this context (low).

##### Treatment in advanced disease (stage IVB)

Patients with metastatic disease or recurrent disease can be treated with EP-based regimens (etoposide and cisplatin) o carboplatin/etoposide (in patients not eligible for cisplatin) for 4 or 6 cycles, or VAC/PE (vincristine, adriamycin, and cyclophosphamide alternated with cisplatin and etoposide) (51, 52). Chemotherapy agents used in the recurrent small cell NEC setting are similar to those used to treat recurrent small cell lung cancer and include single agents such as topotecan, irinotecan, paclitaxel, or docetaxel (51). At the MD Anderson Cancer Center, Salvo et al., suggested triple therapy with topotecan, paclitaxel, and bevacizumab (52).

M. Frumovitz et al., evaluated in a retrospective analysis a cohort comparing the triplet (topotecan, paclitaxel, and bevacizumab) vs. monotherapy. It was shown that the median progression-free survival was 8 months for the triple regimen vs. 4 months for other regimens (HR 0.21; 95% CI: 0.09 to 0.54), and the median overall survival was 9.7 months for the triple regimen and 9.4 months for patients who received other regimens (HR 0.53; 95% CI: 0.23 to 1.22) (53). However, there is no standard treatment for recurrence or progression to EP. Given the rarity of the disease, there is limited consensus on optimal treatment in the recurrent NEC context. Clinical trials and targeted treatment based on molecular profiles should be considered, and treatment should be individualized due to the scarcity of data (54) (**Table 2**).

**Table 2:** Treatment options in recurrent small cell neuroendocrine carcinoma.

Scheme	Study	PFS (months)	OS (months)	Response rate	Other information
TPB (52)	Retrospective	7.8	9.7	77%	Possible treatment period: > 6 months: 62%, > 12 months: 31%
Pembrolizumab (55)	Prospective	2.1	NA	71%	No patient was progression-free at 27 weeks
NIivolumab (56)	Case report	NA	NA	Complete response	Discontinued due to immune-mediated adverse events
Trametinib (57)	Case report	NA	NA	Complete response	KRAS exon 2 mutation

TPB, topotecan, paclitaxel, Bevacizumab; NA, not applicable.

Surveillance and follow-up should consider the possibility of lung, brain, or bone metastases, in addition to the risk of local recurrence. Computed tomography or magnetic resonance imaging of the brain should be considered in case of neurological symptoms, changes in mental status, or lung metastases (51).

#### Treatment in advanced disease (Stage IVB)

##### Recommendation

• First-line systemic chemotherapy with the etoposide/cisplatin or etoposide/carboplatin scheme for 6 cycles is the standard for distant metastasis (high).
• In cases of progression or recurrence, there is no standard treatment. For a platinum-free interval >6 months, reconsider EP scheme; for <6 months, consider monotherapy with topotecan, irinotecan, paclitaxel, or docetaxel (moderate).
• Targeted therapies or immunotherapy are recommended under protocols or clinical trials.

## Conclusions

This mini-review highlights that rare cervical histologies, including small cell carcinoma, mucinous gastric-type adenocarcinoma, clear cell adenocarcinoma, and adenosquamous (glassy cell) variants, exhibit aggressive behavior. Such varieties show deleterious responses to treatment and consequently a worse survival prognosis. Molecular studies currently identify potential molecular targets for treatment. However, despite some case reports indicating favorable responses, immune checkpoint inhibitors have so far shown limited benefit in these histological varieties. While new treatment strategies are becoming more common in squamous histological cervical cancer, recruiting patients for prospective studies in these histological varieties remains a significant challenge. Decision-making relies on information derived from retrospective reviews, case reports, and a few phase II clinical trials, especially in neuroendocrine histologies. Therefore, there is a need to implement clinical trials through multicenter and multinational collaborations. The Clinical Trial Reporting Program led by the M.D. Anderson Cancer Center, titled “Establishing a Tumor Registry for Patients With Neuroendocrine Carcinoma of the Cervix (NeCTuR)” (https://clinicaltrials.gov/ct2/show/NCT0472309), aims to prospectively and retrospectively collect data on disease characterization, treatment, and outcomes for patients with neuroendocrine carcinoma of the uterine cervix.

## Author contributions

EA-B: Conceptualization, Supervision, Writing – original draft, Writing – review & editing. TG-C: Writing – original draft, Writing – review & editing. LC-P: Writing – original draft, Writing – review & editing. CF-T: Writing – original draft, Writing – review & editing. ME-A: Writing – original draft, Writing – review & editing. JG-P: Writing – original draft, Writing – review & editing. EG-G: Writing – original draft, Writing – review & editing.
